# Gastrodin Alleviates Post-Stroke Headache and Neuronal Injury via Activating the PI3K/Akt Pathway

**DOI:** 10.1007/s11064-026-04932-2

**Published:** 2026-11-12

**Authors:** Yanping Ai, Tongmei Zhang, Tao Wang, Quntao Yu, Hua Huang, Wenyong Gao, Shufang Hu

**Affiliations:** 1 Department of Neurology, Wuhan Hankou Hospital, Wuhan University of Science and Technology, Wuhan, 430000 China; 2 Department of Histology and Embryology, School of Basic Medicine and Tongji Medical College, Huazhong University of Science and Technology, Wuhan, 430030 China; 3 Department of Urology, Wuhan Hankou Hospital, Wuhan University of Science and Technology, Wuhan, 430000 China; 4 Department of Endocrinology, Wuhan Hankou Hospital, Wuhan University of Science and Technology, Wuhan, 430000 China

**Keywords:** Gastrodin, Acute ischemic stroke, PI3K/Akt signaling pathway, Post-stroke headache

## Abstract

Acute ischemic stroke (AIS) is frequently accompanied by severe neurological deficits and post-stroke headache (PSH). These complications significantly worsen clinical outcomes. Although gastrodin has been reported to exert neuroprotective effects, whether it relieves PSH and the underlying mechanisms remain unclear. In this study, using a rat model of PSH that exhibits orbital mechanical allodynia and increased facial grooming, we demonstrated that gastrodin dose-dependently alleviated pain-related behaviors, reduced the number of c-Fos-positive neurons in the trigeminal nucleus caudalis, and decreased protein levels of calcitonin gene-related peptide (CGRP) and substance P in the trigeminal ganglion. Mechanistically, gastrodin restored PI3K/Akt pathway activity in the ischemic penumbra, as reflected by increased PI3K and Akt expression and a higher p-Akt/Akt ratio, which was accompanied by reduced cerebral infarct volume, alleviated neuronal pathological damage, and suppressed oxidative stress, inflammation, and apoptosis. All these protective effects were significantly reversed by the PI3K inhibitor LY294002. To validate clinical relevance, we enrolled 80 AIS patients and 50 healthy controls. Peripheral blood PI3K/Akt expression was significantly lower in AIS patients and was correlated with PSH severity and neurological deficits. In conclusion, we revealed that gastrodin exerts dual analgesic and neuroprotective effects by restoring the PI3K/Akt pathway, providing novel insights and experimental support for the treatment of AIS complicated with PSH.

**Graphical Abstract:**

**Figure:**
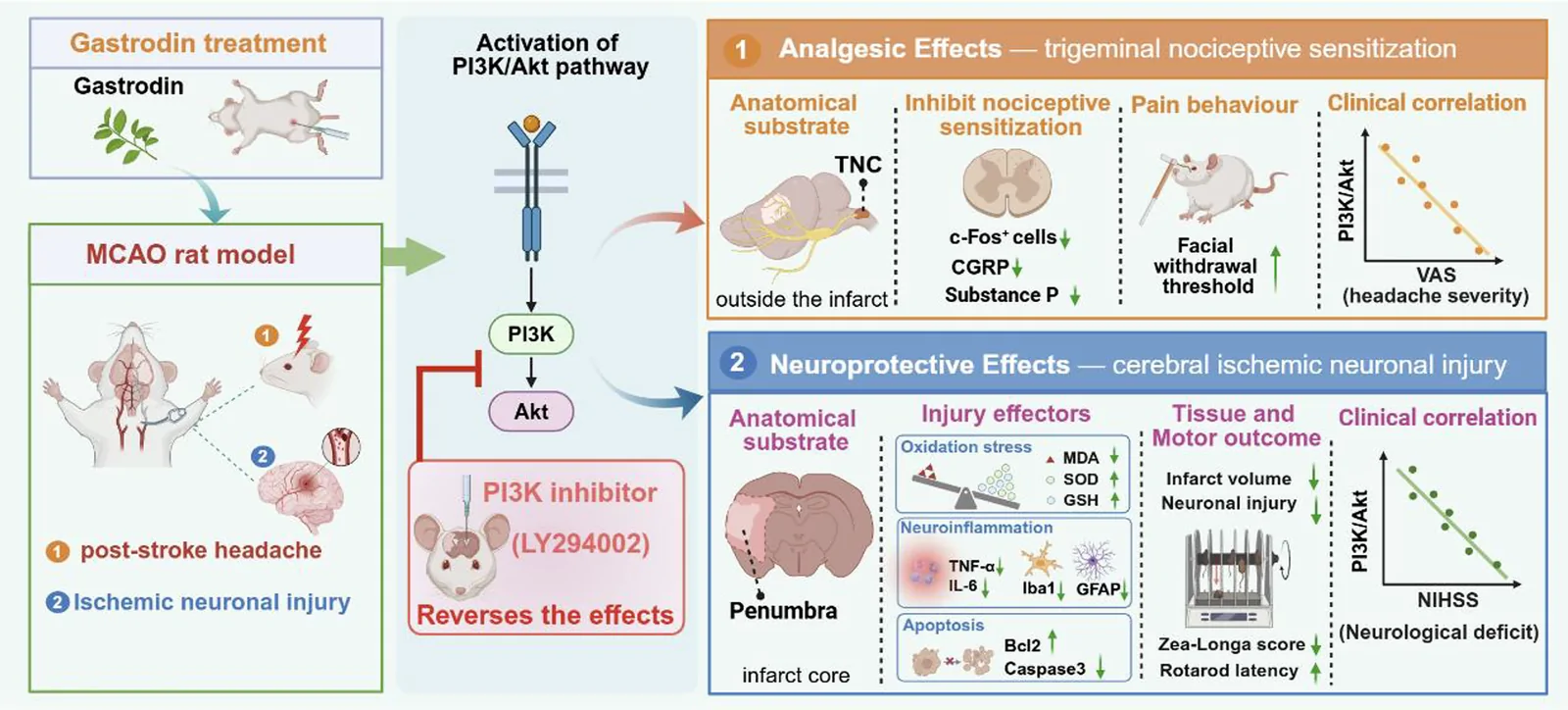

**Supplementary Information:**

The online version contains supplementary material available at https://doi.org/10.1007/s11064-026-04932-2.

## Introduction

Acute Ischemic Stroke (AIS) is a sudden neurological deficit syndrome caused by cerebral artery occlusion and is the leading cause of adult mortality and long-term disability worldwide [1, 2]. Recent data from the Global Burden of Disease Study showed that there were approximately 11.9 million new stroke cases globally in 2021, of which ischemic stroke accounting for 65.3%. Its high incidence, mortality, and disability rates impose a heavy burden on public health systems, socioeconomics, and patients’ families [3].

The pathophysiology of AIS is complex and involves multiple cascades triggered rapidly after cerebral blood flow interruption, including excitatory amino acid toxicity, mitochondrial damage-driven oxidative stress, excessive neuroinflammation, and blood–brain barrier disruption. These events cause rapid and irreversible necrosis of neurons in the infarct core and progressive damage in the surrounding ischemic penumbra, ultimately leading to persistent neurological deficits manifested as motor dysfunction, sensory abnormalities, cognitive decline, and speech impairment [4–6]. In addition to primary neurological deficits, various post-stroke complications severely hinder rehabilitation and reduce quality of life. Among them, post-stroke headache (PSH) is one of the most common yet underrecognized complications [7, 8]. Epidemiological data indicate that the incidence of acute headaches in AIS patients ranges from 15% to 40%, and 1% to 23% of stroke survivors develop chronic PSH lasting more than 3 months. PSH not only causes physical discomfort and psychological stress but is also closely associated with poor neurological recovery, increased depression risk, and reduced long-term quality of life [9, 10]. The precise pathogenesis of PSH remains incompletely understood. Current studies suggest that infarcts may directly or indirectly activate the trigeminal vascular system (TVS), inducing abnormal release of algogenic neuropeptides such as calcitonin gene-related peptide (CGRP) and substance P, thereby triggering central sensitization and headache [11–14]. Notably, neurons in the ischemic penumbra retain reversible survival potential in the early stage of stroke [15], providing a critical therapeutic window for neuroprotection. Although ischemic neuronal injury and PSH are currently studied as separate entities, emerging evidence suggests they may share common molecular substrates [9]. Both conditions converge on signaling cascades governing neuronal survival, oxidative stress, neuroinflammation, and synaptic excitability. The trigeminal vascular system, which is activated by algogenic neuropeptides released from the ischemic penumbra, may serve as a direct mechanistic bridge linking infarct and headache generation [16]. Identifying a pathway that simultaneously regulates ischemic damage and trigeminal nociceptive sensitization would therefore provide a mechanistic basis for treating both the neurological deficit and the headache complication of AIS [17]. Therefore, there is an urgent need to develop novel strategies that simultaneously protect penumbral neurons, alleviate core neurological deficits, and address complications such as PSH in cerebrovascular disease research.

The Phosphoinositide 3-kinase/Protein Kinase B (PI3K/Akt) signaling pathway is a central intracellular signaling hub that widely regulates cell growth, proliferation, metabolism, and survival [18, 19]. This pathway is increasingly recognized as a key player in AIS pathophysiology [20, 21]. Numerous preclinical studies have confirmed that multiple growth factors, cytokines, and neurotransmitters activate receptor tyrosine kinases and initiate the PI3K/Akt pathway under cerebral ischemia or hypoxia, exerting multifaceted neuroprotection [19, 22, 23]. On the one hand, Akt inhibits pro-inflammatory pathways such as NF-κB, reducing the production and release of TNF-α, IL-6, and other inflammatory cytokines, thereby suppressing excessive neuroinflammation [24]. On the other hand, Akt activates Nrf2, a master transcription factor of the antioxidant system, which upregulates a series of antioxidant enzymes to counteract oxidative stress injury [25]. Furthermore, PI3K/Akt activation directly inhibits caspase-mediated apoptotic cascades by balancing Bcl-2/Bax family proteins and modulates autophagy via the mTOR pathway [26]. Despite robust preclinical evidence for PI3K/Akt-mediated neuroprotection in experimental stroke [19, 22, 23], its role in PSH remains unexplored. Emerging data, however, indicate that PI3K/Akt is not solely a neuronal survival pathway but also a regulator of nociceptive signaling and central sensitization in the trigeminal system. In a rat model of vestibular migraine, PI3K/Akt activation in the trigeminal nucleus caudalis (TNC) and vestibular nuclei accompanied elevated pro-inflammatory cytokines and increased expression of the central sensitization markers CGRP and c-Fos; CXCL10 knockdown suppressed PI3K/Akt activation and attenuated these changes together with pain hypersensitivity, and the PI3K/Akt activator 740 Y-P partially reversed these effects [27]. Similarly, in another vestibular migraine model, TREM2 promoted neuroinflammation and central sensitization in the trigeminocervical complex and vestibular nuclei through PI3K/Akt/mTOR-mediated suppression of autophagy, and TREM2 inhibition or autophagy activation reversed these alterations [17]. These findings position PI3K/Akt at the intersection of two processes that have traditionally been studied in isolation: neuronal survival after cerebral ischemia and nociceptive sensitization in the trigeminal system. However, whether this pathway acts as a shared mechanistic link under the specific condition of AIS, in which ischemic injury and PSH occur simultaneously, has not been directly examined. Its expression profile in AIS patients and clinical associations with the severity of neurological deficits as well as PSH are still unclear.

In traditional Chinese medicine theory, ischemic stroke falls into the categories of “stroke” or “cerebral infarction,” with the core pathogenesis being yin-yang imbalance and qi-blood disharmony in the internal organs, and wind, fire, phlegm, and stasis obstructing the cerebral collaterals [28]. *Gastrodia elata* Blume (Tianma) is sweet in taste, neutral in nature, and acts on the liver meridian. It has long been regarded as a key herb for calming liver wind, unblocking collaterals, and relieving pain, and is widely used for stroke hemiplegia, dizziness, and headache [29]. Gastrodin, the major phenolic glycoside monomer extracted from *Gastrodia elata* tubers, has a small molecular weight and high liposolubility, enabling it to easily cross the blood–brain barrier. In recent years, it has shown excellent pharmacological activity in cerebrovascular disease research [30, 31]. Moreover, gastrodin exhibits significant pharmacological activity in headache-related disorders and is now widely used in the clinical treatment of migraine. Animal studies have also confirmed that gastrodin and *Gastrodia* extracts can inhibit the expression and release of CGRP in the trigeminal ganglion, demonstrating good analgesic potential [32]. Although previous literature has reported that gastrodin exerts neuroprotective effects by activating the PI3K/Akt pathway [33], whether it can simultaneously relieve post-stroke headache through the same pathway, and the clinical correlation between PI3K/Akt pathway inhibition and PSH, have not been systematically investigated. Gastrodin is particularly suitable for testing this hypothesis because it has been reported both to activate PI3K/Akt and to inhibit CGRP release in the trigeminal system. If PI3K/Akt indeed serves as a common pathway linking ischemic injury to trigeminal nociceptive sensitization, gastrodin should produce parallel neuroprotective and analgesic effects, and both effects should be abolished by pharmacological inhibition of PI3K/Akt. This prediction provides the rationale for the experimental design of the present study.

We therefore hypothesized that PI3K/Akt serves as a common pathway linking cerebral ischemic damage to trigeminal nociceptive sensitization, and that restoring PI3K/Akt pathway activity confers both neuroprotection and PSH relief. To test this hypothesis, we designed three complementary arms: (i) in an MCAO-induced PSH rat model, we determined whether gastrodin produces parallel neuroprotective and analgesic effects and whether both are reversed by the PI3K inhibitor LY294002; (ii) we dissected the downstream mechanisms, including oxidative stress, neuroinflammation, apoptosis, and trigeminal nociceptive activation; and (iii) in AIS patients, we tested whether peripheral blood PI3K/Akt expression is downregulated and correlates with neurological deficit and headache severity. This design directly tests PI3K/Akt as a shared mechanistic link between ischemic brain injury and PSH, and supports gastrodin as a dual-action therapeutic agent.

## Materials and Methods

### Reagents and Antibodies

Reagents and antibodies used in this study were obtained from the indicated sources: Monofilament nylon suture (Beijing Xinnong, 3043-A4); gastrodin (Aladdin, G299059); ELISA kits for rat malondialdehyde (Elabscience, E-BC-K025-M), superoxide dismutase (Elabscience, E-BC-K020-M), glutathione (Elabscience, E-EL-0026), TNF-α (Elabscience, E-EL-R2856), IL-6 (Elabscience, E-EL-R0015), human PI3K (ELK biotechnology, ELK9879), and human Akt (ELK biotechnology, ELK0371); PI3K inhibitor LY294002 (MedChemExpress, USA); TTC staining solution (biosharp, BL1215A); Nissl staining solution (biosharp, BL1039A); hematoxylin-eosin staining solution (Hubei Pumei Biotechnology, PMK0270); Trizol (Invitrogen, 15596018); reverse transcription kit (Vazyme, R233-01); qPCR kit (Vazyme, Q712-02); RIPA lysis buffer (Beyotime, Shanghai); primer synthesis (Sangon, Shanghai); Primary antibodies against c-Fos (Synaptic Systems Antibody, Cat# 226017, RRID: AB_2864765; IF 1:200), caspase-3 (Proteintech, Cat# 19677-1-AP, RRID: AB_10733244; IF 1:150, WB 1:800), Bcl-2 (Proteintech, Cat# 68103-1-Ig, RRID: AB_2923635; IF 1:200; WB 1:1000), PI3K (Proteintech, Cat# 60225-1-IG, RRID: AB_11042594; WB 1:800), Akt (Proteintech, Cat# 60203-2-Ig, RRID: AB_10912803; WB 1:1000), p-Akt (Proteintech, Cat# 66444-1-Ig, RRID: AB_2782958; WB 1:1000), CGRP (ABclonal, Cat# A5542, RRID: AB_2766323; WB 1:800), substance P (Invitrogen, Cat# PA5-106934, RRID: AB_2854598; WB 1:1000), Iba1(Wako, Cat# 019–19741, RRID: AB_839504; IF 1:300), GFAP (Cell signaling Technology, Cat# 3670 S, RRID: AB_561049; IF 1:200), and GAPDH (Proteintech, Cat# 60004-1-Ig, RRID: AB_2107436; WB 1:3000); Donkey Anti-Rat IgG H&L (Alexa Fluor 555) preadsorbed (Abcam, Cat# ab150154, RRID: AB_2813834; dilution ratio 1:350), Alexa Fluor 488- and 546-conjugated secondary antibodies (Invitrogen, Cat# A-21202, RRID: AB_141607; Cat# A10040, RRID: AB_2534016; dilution ratio 1:200) for Immunofluorescence; IRDye^®^ 800CW Goat anti- Mouse and Goat Anti-Rabbit IgG Secondary Antibody for western blot (LI-COR, Cat# 926–32210, RRID: AB_621842; Cat# 926–32211, RRID: AB_621843; dilution ratio 1:10000).

### Experimental Animals

Male Sprague-Dawley (SD) rats (weight 250–280 g, SPF grade) were purchased from the Experimental Animal Center of Three Gorges University. Rats were housed in an independent ventilation cage (IVC) system under controlled temperature (22 ± 2 °C), humidity (50%−60%), and a 12-hour light/dark cycle, with free access to food and water. All animal experiments were approved by the Animal Ethics Committee, and rats were acclimated for one week before modeling.

### MCAO Model Establishment and Intervention

Except for the Sham group, rats in all other groups were subjected to left middle cerebral artery occlusion (MCAO) [34, 35]. Prior to surgery, rats were fasted for 12 h, followed by intraperitoneal injection of 1.25% tribromoethanol (Avertin; dosage: 10 mL/kg; Nanjing Aibei Biotechnology) to minimize pain and discomfort during the surgical procedure [36]. Atropine was then administered subcutaneously at a dose of 0.1 mg/kg to maintain unobstructed respiratory tracts. After anesthesia, rats were fixed in the supine position, and a midline cervical incision was created to isolate the left common carotid artery (CCA), external carotid artery (ECA), and internal carotid artery (ICA). The ECA was ligated, and a silicon-tipped nylon monofilament suture was slowly advanced into the ICA via the proximal ECA to block blood flow to the middle cerebral artery. The cervical incision was sutured upon surgery completion, and the wound together with the surrounding tissue within a minimum 1.5 cm radius was thoroughly disinfected with iodophor. Rats in the sham group underwent identical surgical procedures without insertion of the occluding suture. Cerebral blood flow was monitored by laser speckle flowmetry after surgery. Successful ischemic modeling was defined as a reduction in blood flow on the ischemic hemisphere exceeding 45% ± 10% relative to baseline perfusion [37]. All rats were individually placed in a thermostatted container maintained at 37 °C until complete recovery from anesthesia. Functional jelly gel (Shulaibao Biotechnology, Cat. No. SL0042) was placed in each cage to facilitate food and water access for hemiplegic animals.

On the day after MCAO surgery, rats presenting post-stroke headache (PSH)-like phenotypes were screened and randomly assigned to five groups: PSH model group (PSH + PBS), low-dose gastrodin group (PSH + Gas-L, 50 mg/kg), medium-dose gastrodin group (PSH + Gas-M, 100 mg/kg), high-dose gastrodin group (PSH + Gas-H, 200 mg/kg), and high-dose gastrodin combined with PI3K inhibitor group (PSH + Gas-H + LY). Gastrodin was dissolved in normal saline and administered intraperitoneally once daily for 21 days; the doses were selected based on previous reports in cerebral ischemia models [38, 39]. To avoid exacerbating brain injury on the ischemic side and ensure adequate drug diffusion, rats in the PSH + Gas-H + LY group received intracerebroventricular injection of LY294002 (10 µM, 10 µL) into the right lateral ventricle 30 min prior to gastrodin administration, three times weekly for three consecutive weeks. Rats in the sham and PSH + PBS groups received daily intraperitoneal injection of an equal volume of normal saline for a total of 21 days of intervention.

### Laser Speckle Blood Flow Imaging

Anesthetized rats were placed on a thermostatted operating table. After scalp preparation, a midline incision was made along the cranial vertex. The subcutaneous tissue and periosteal fascia were bluntly dissected to fully expose the calvarium over both the left and right hemispheres, and the skull was polished with a cranial drill while maintaining a clean and intact bone surface. The rat’s head was secured to the dedicated positioning bracket of the laser speckle blood flow imaging system. The height and angle of the imaging probe were adjusted to fully cover the blood supply territories of cerebral cortices, avoiding cranial sutures and vascular shadows. The instrument was warmed up, imaging parameters were set, and real-time speckle images of local cerebral blood flow were continuously acquired under dark, quiet, vibration- and light-proof conditions. After imaging, the region of interest was selected using the accompanying analysis software to quantitatively measure and statistically analyze changes in local cerebral blood flow before and after modeling, thus evaluating cerebral ischemic perfusion defects in the MCAO model.

### Behavioral Testing

Twenty-four hours after MCAO surgery, neurological function was assessed using the Zea-Longa score (0–4) [40]: 0, no deficit; 1, failure to fully extend the right forepaw (mild focal deficit); 2, circling to the (moderate focal deficit); 3, falling to the right (severe deficit); 4, unable to walk spontaneously, decreased level of consciousness. In addition to the CBF criterion, the Zea-Longa score was used to evaluate neurological function at 24 h after MCAO. Rats were considered successfully modeled only if they met both of the following criteria: (i) a reduction in ipsilateral cerebral blood flow exceeding 45% ± 10% of baseline perfusion, as monitored by laser speckle flowmetry, and (ii) a Zea-Longa score of 1–3 at 24 h. Rats were excluded before PSH screening if any of the following applied: (i) failure to reach the predefined CBF reduction threshold; (ii) a Zea-Longa score of 0 or 4 at 24 h; (iii) death within 24 h after surgery; or (iv) failure of suture placement during surgery. The number of rats with successful modeling and the mortality at each stage are summarized in Table S1 and Table S2, respectively. After the 21-day treatment period, the Zea-Longa score was assessed again to evaluate the effects of different treatments on neurological function.

For post-stroke headache-like behaviors [41], rats were monitored in home cages for 4 h, and the time spent on facial grooming was recorded. To assess periorbital mechanical allodynia (mimicking migraine-related pain responses), von Frey filaments were used to measure the mechanical withdrawal threshold of the face. One day before testing, periorbital and forehead skin was shaved to avoid interference. Rats were then placed individually in ventilated behavioral test chambers and allowed to acclimate for 30 min. During testing, calibrated filaments were applied perpendicularly to the shaved skin with gradually increasing force until the filament bent, maintaining the pressure for 5 s. A positive response was defined as rapid facial grooming, paw scratching of the stimulated site, head shaking, or active head withdrawal. Filament forces ranged from 1.0 to 15 g (1.0, 1.4, 2.0, 4.0, 6.0, 8.0, 10, 15 g). Each intensity was applied three times with an interval of at least 2 s between stimulations. The threshold was the force at which three positive responses were observed.

All behavioral assessments, including the Zea-Longa score and von Frey filament testing, were performed by an investigator blinded to the group allocation. Animals were identified by coded numbers, and treatment information was concealed throughout testing and scoring. Data analysis was also performed with the analyst blinded to group identity.

### TTC Staining

After behavioral assessments, randomly selected rats were deeply anesthetized and decapitated; brains were rapidly removed and frozen at −20 °C for 20 min. Coronal brain slices (2 mm thick) were completely immersed in 2% TTC solution and incubated at 37 °C in the dark for 30 min, then fixed in 4% paraformaldehyde for 24 h. Infarct volume was calculated using ImageJ software, corrected for edema using the formula: Infarct volume (%) = [(Contralateral hemisphere volume - Ipsilateral non-infarcted volume)/(Contralateral hemisphere volume × 2)] × 100%.

### Histological Staining

After cardiac perfusion and fixation with 4% paraformaldehyde, rat brains were dehydrated in 15% and 30% sucrose gradients. After sinking, frozen sections were cut. Coronal sections from the ischemic penumbra were stained with hematoxylin-eosin (HE) to observe overall tissue morphology and necrosis, and with Nissl staining to specifically label Nissl bodies for evaluating neuronal structural integrity and density.

Frozen brain sections were permeabilized with 0.5% Triton X-100 for 15 min and washed three times with PBS containing 0.1% Triton X-100. Sections were then blocked with 3% bovine serum albumin (BSA) for 1 h at room temperature, followed by incubation with primary antibodies overnight at 4 °C. After three washes with PBS containing 0.1% Triton X-100, sections were incubated with the corresponding fluorescent secondary antibodies for 1.5 h at room temperature and counterstained with DAPI for nuclear labeling. Images were captured using a confocal laser scanning microscope.

### Western Blot

Total protein was extracted from ischemic penumbra brain tissue and quantified by BCA assay. Equal amounts of protein were separated by SDS-PAGE and transferred to PVDF membranes. After blocking with 5% non-fat milk for 1 h at room temperature, membranes were incubated with primary antibodies against PI3K, Akt, p-Akt, Caspase-3, Bcl-2, CGRP, substance P, and GAPDH overnight at 4 °C. After washing with TBST, membranes were incubated with secondary antibodies for 1.5 h at room temperature. Protein bands were visualized using a two-color infrared laser scanning imaging system (Odyssey CLX), and band gray values were semiquantitatively analyzed using ImageJ software.

### Clinical Subjects and Grouping

This study was approved by the Ethics Committee of Wuhan Hankou Hospital (approval number: hyll2024006), and written informed consent was obtained from all subjects. Eighty patients with acute ischemic stroke (AIS) admitted during a specific study period were prospectively enrolled as the AIS group. Inclusion criteria: (1) meeting the diagnostic criteria of the *Chinese Guidelines for the Diagnosis and Treatment of Acute Ischemic Stroke* and confirmed by brain MRI or CT imaging; (2) onset to admission time ≤ 72 h; (3) presence of definite focal neurological deficits or accompanying new-onset headache symptoms. Exclusion criteria: concomitant hemorrhagic stroke, brain tumor, or severe intracranial infection; severe heart, liver, kidney, or other vital organ failure; severe mental disorders or cognitive deficits that precluded evaluation. During the same period, 50 age- and sex-matched healthy volunteers were recruited as the normal control group.

### Clinical Scale Assessment

At admission and follow-up visits, two experienced physicians performed blinded scale assessments. The National Institutes of Health Stroke Scale (NIHSS) (Cronbach’s α = 0.79, ICC = 0.85, range 0–42, higher scores indicate more severe neurological impairment) was used to assess neurological deficits. The Visual Analog Scale (VAS) (range 0–10) was used to quantify headache intensity.

### RT-qPCR and ELISA

Peripheral venous blood (5 mL) was collected from each subject into EDTA-K2 anticoagulant vacuum tubes. Plasma and peripheral blood mononuclear cells (PBMCs) were separated. Total RNA was extracted from PBMCs of human subjects using Trizol, and after purity and concentration assessment by UV spectrophotometry, reverse transcription was performed to obtain cDNA. Real-time quantitative PCR was carried out using SYBR Green. Cycling conditions: 95 °C for 5 min, followed by 40 cycles of 95 °C for 10 s, 60 °C for 30 s. GAPDH was used as an internal control, and relative mRNA expression levels of PI3K and Akt were calculated using the 2^-ΔΔCt method. qPCR was performed using the following primers: PI3K: 5′-ACTTCCCCTGTCAGATCCAT-3′ (forward) and 5′- AACTCCTTCCACCACCAGAT-3′ (reverse); Akt: 5′- GCTGGACGATAGCTTGGA-3′ (forward) and 5′-CTCACGTTGTCCAGGTCA-3′ (reverse); GAPDH: 5′- GTCTCCTCTGACTTCAACAGCG-3′ (forward) and 5′- ACCACCCTGTTGCTGTAGCCAA-3′ (reverse). 

In addition, following the manufacturer’s instructions of ELISA kits, we measured superoxide dismutase (SOD) activity, malondialdehyde (MDA) and reduced glutathione (GSH) content, and the release levels of pro-inflammatory cytokines TNF-α, and IL-6 in rat ischemic penumbra brain tissue homogenates, as well as plasma PI3K and Akt protein concentrations in human subjects.

### Statistical Analysis

Data analysis was performed using SPSS 26.0. Normally distributed continuous data were expressed as mean ± standard error of the mean (SEM). Comparisons among multiple groups were performed using one-way ANOVA followed by Tukey’s post hoc test. Dose-response among the PSH + PBS, Gas-L, Gas-M and Gas-H groups was assessed by one-way ANOVA with a post-test for linear trend; a dose-dependent effect was claimed when *P* for trend < 0.05 (Table S3). Correlation between clinical gene expression and clinical scores was assessed using Pearson correlation analysis. To evaluate the diagnostic efficacy of PI3K/Akt pathway components for severe PSH, patients were post hoc dichotomized into severe (VAS ≥ 7) and non-severe (VAS < 7) subgroups based on a clinically established pain-severity threshold, and receiver operating characteristic (ROC) curve analysis was performed for PI3K mRNA, PI3K protein, Akt mRNA, and Akt protein. The area under the curve (AUC), 95% confidence interval, optimal cut-off value, sensitivity, and specificity were calculated for each marker. *P* < 0.05 was considered statistically significant.

## Results

### Gastrodin Alleviates Post-Stroke Headache-Like Behaviors and Suppresses Trigeminal Nociceptive Activation in MCAO Rats

**Fig. 1:**
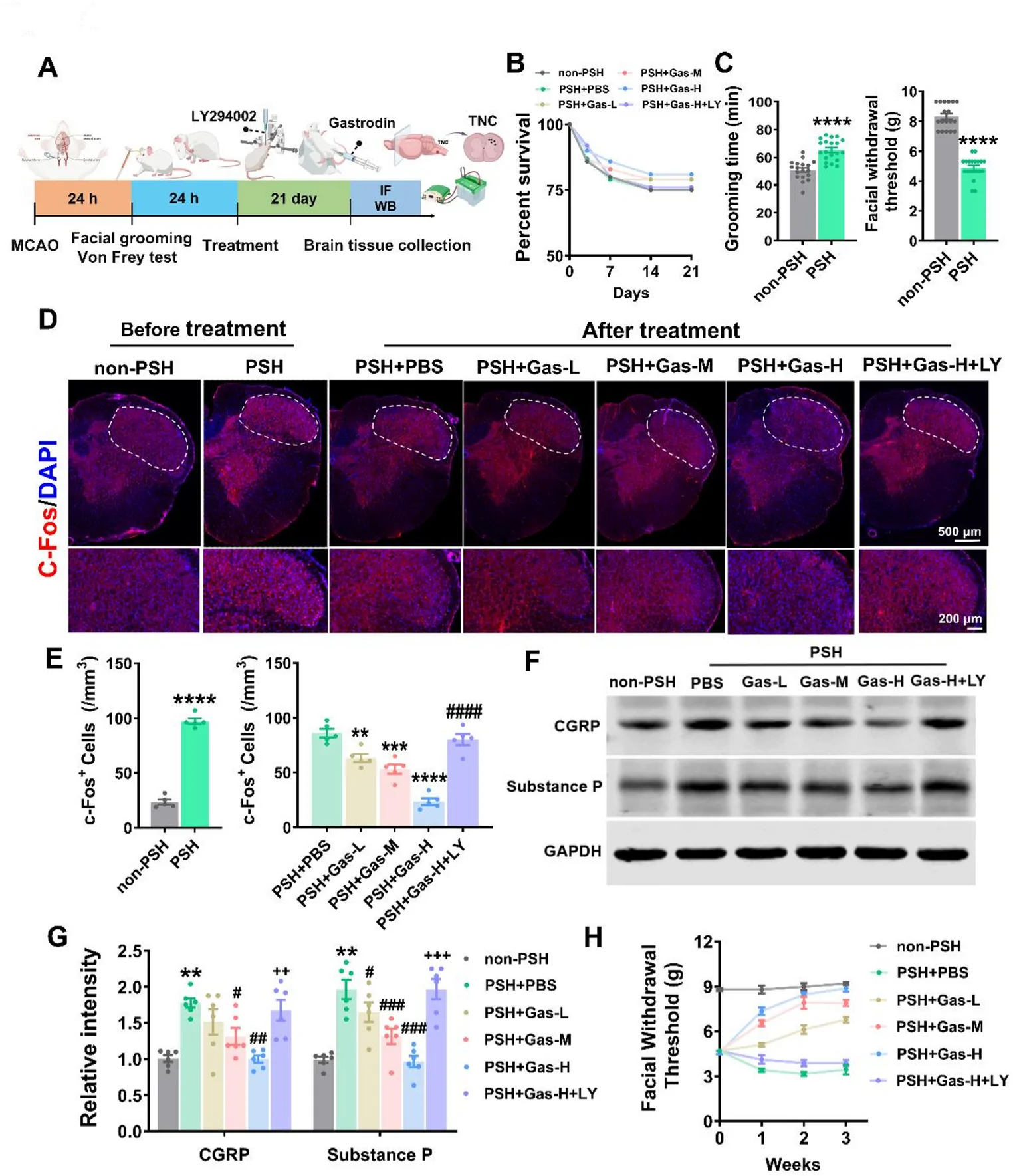
Gastrodin attenuates post-stroke headache-like behaviors and trigeminal nociceptive activation in MCAO rats. **A** Schematic of the experimental timeline. **B** Survival curves of rats in each group during the 21-day treatment period. **C** Quantification of facial grooming time (left panel) and periorbital mechanical withdrawal threshold (right panel). **D** Representative immunofluorescence images of c-Fos (red) and DAPI (blue) in the trigeminal nucleus caudalis (TNC). **E** Quantification of c-Fos-positive cells in the TNC. *N* = 5 rats, with 5 brain slices collected from each animal for quantification and then averaged per rat. Data are presented as mean ± SEM. ****P < 0.0001 vs. non-PSH group (left); **P < 0.01, ***P < 0.001, ****P < 0.0001 vs. PSH-PBS group; ^####^*P* < 0.0001 vs. PSH-Gas-H group (right). Statistical comparisons were performed by one-way ANOVA followed by Tukey’s post hoc test. **F** Representative Western blot bands of substance P, CGRP, and GAPDH in the trigeminal ganglion. **G** Quantification of substance P and CGRP protein levels normalized to GAPDH. *N* = 6 rats/group. Data are presented as mean ± SEM. **P < 0.01 vs. non-PSH group; ###*P* < 0.001 vs. PSH + PBS group; ++*P* < 0.01, +++*P* < 0.001 vs. PSH-Gas-H group. Statistical comparisons were performed by one-way ANOVA followed by Tukey’s post hoc test. **H** Dynamic monitoring of periorbital mechanical withdrawal threshold over 3 weeks of treatment

To investigate the therapeutic effect of gastrodin (Gas) on post-stroke headache (PSH) induced by middle cerebral artery occlusion (MCAO), a left MCAO rat model was established in the present study. A total of 173 rats were used, of which 155 underwent MCAO surgery; 133 were successfully modeled (85.81% of the operated rats; Table S1). At 24 h after MCAO surgery, rats presenting mechanical allodynia and increased spontaneous facial grooming were screened as PSH-positive animals via facial grooming behavioral observation and periorbital Von Frey filament test. Subsequently, the screened PSH rats received 21 consecutive days of gastrodin treatment, and the detailed experimental timeline is illustrated in Fig. 1A. The 21-day survival curves of rats in each group are presented in Fig. 1B, with detailed mortality data summarized in Table S2. Behavioral tests revealed that compared with the non-PSH blank control group, PSH model rats exhibited markedly prolonged total facial grooming time and drastically decreased periorbital mechanical withdrawal threshold (Fig. 1C, *P* < 0.0001), confirming the successful establishment of the PSH model.

Immunofluorescence staining for c-Fos in the caudal subnucleus of the trigeminal spinal tract (TNC) demonstrated that the number of c-Fos-positive neurons in the TNC was significantly elevated in PSH rats relative to non-PSH controls, whereas gastrodin administration reduced the abundance of c-Fos⁺ cells in a dose-dependent manner (Fig. 1D and E; *P* for trend < 0.0001). Further western blot analysis illustrated that gastrodin intervention dose-dependently downregulated the protein levels of two pivotal pro-nociceptive neuropeptides, calcitonin gene-related peptide (CGRP) and substance P, in the trigeminal ganglion of PSH rats (Fig. 1F and G; *P* for trend < 0.0001 for both). Notably, co-administration of the PI3K inhibitor LY294002 fully reversed the inhibitory effects of gastrodin on c-Fos expression, CGRP and substance P production (Fig. 1D-G). Dynamic monitoring of pain indicators throughout the entire 21-day treatment period showed that the mechanical withdrawal threshold recovered in a dose-dependent manner in the Gas-L, Gas-M and Gas-H groups (Fig. 1H; *P* for trend < 0.0001 at week 3), with the effect approaching a plateau between 100 and 200 mg/kg (non-linear component *P* = 0.0002). In contrast, intracerebroventricular pre-injection of LY294002 abolished the anti-allodynic effect of high-dose gastrodin (Fig. 1H). These results demonstrate that high-dose gastrodin effectively alleviates PSH-like behaviors in MCAO rats and suppresses aberrant activation of the trigeminal nociceptive pathway in a PI3K/Akt signaling pathway-dependent manner.

### Gastrodin Improves Neurological Deficits and Reduces Cerebral Infarct Volume in PSH Rats

**Fig. 2:**
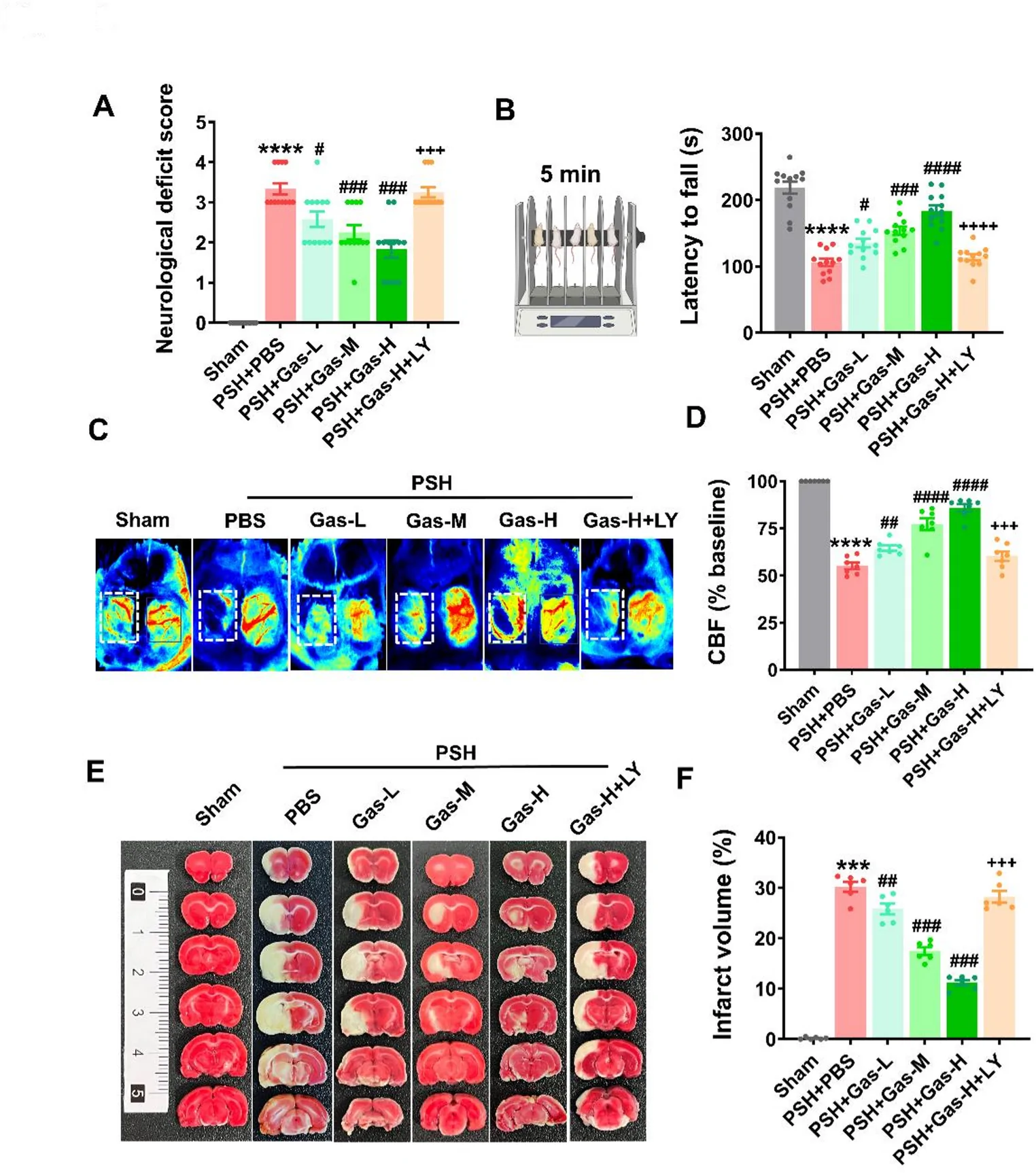
Gastrodin improves neurological deficits and reduces cerebral infarct volume in PSH rats via the PI3K/Akt pathway. **A** Neurological deficit scores assessed by the Zea-Longa Score after 21 days of treatment. Groups: Sham, PSH + PBS, PSH + Gas-L (50 mg/kg), PSH + Gas-M (100 mg/kg), PSH + Gas-H (200 mg/kg), and PSH + Gas-H + LY (200 mg/kg gastrodin + LY294002). *N* = 12 rats/group. **B** Latency to fall in the rotarod test. *N* = 12 rats/group. **C** Representative laser speckle images of cerebral blood flow (CBF) in each group. Dashed boxes indicate the regions of interest. **D** Quantitative analysis of CBF presented as the percentage of baseline in each group. *N* = 7 rats/group. **E** Representative 2,3,5-triphenyltetrazolium chloride (TTC) staining of coronal brain sections from each group. White areas indicate infarcted tissue. **F** Quantification of infarct volume. Data are presented as mean ± SEM. ** *P* < 0.01, *** *P* < 0.001 vs. Sham; ^###^*P* < 0.001 vs. PSH + PBS group; ^+++^*P* < 0.001, ^++++^*P* < 0.0001 vs. PSH + Gas-H group. Statistical comparisons were performed by one-way ANOVA followed by Tukey’s post hoc test

To evaluate the neuroprotective effect of gastrodin against cerebral ischemic injury, multi-dimensional assessments were performed on MCAO-induced PSH rats. Compared with the Sham group, the PSH model group (PSH + PBS) exhibited significant neurological deficits (high Zea-Longa scores, Fig. 2A), impaired motor coordination (decreased rotarod latency, Fig. 2B), and a marked reduction in cerebral blood flow (CBF, Fig. 2C and D). TTC staining confirmed the presence of large cerebral infarcts in the model group (Fig. 2E and F).

After 21 days of intraperitoneal treatment with gastrodin (50, 100, 200 mg/kg), all treatment groups (PSH + Gas-L, PSH + Gas-M, PSH + Gas-H) showed significantly decreased Zea-Longa scores and increased rotarod latency in a dose-dependent manner compared with the PSH model group (Fig. 2A and B; *P* for trend < 0.0001). Gastrodin treatment also significantly restored CBF in a dose-dependent manner (Fig. 2C and D; *P* for trend < 0.0001), and dose-dependently reduced the infarct volume (Fig. 2E and F; *P* for trend < 0.0001). Co-administration of the PI3K inhibitor LY294002 significantly antagonized the neuroprotective effects induced by high-dose gastrodin, as evidenced by increased Zea-Longa scores, decreased rotarod latency, reduced CBF, and increased infarct volume (Fig. 2A-F). These results indicate that gastrodin improves cerebral perfusion and reduces cerebral infarction through enhancement of the PI3K/Akt pathway, thereby effectively alleviating neurological deficits in PSH rats.

### Gastrodin Attenuates Neuronal Pathological Damage in the Ischemic Penumbra and Suppresses Oxidative Stress and Neuroinflammation

**Fig. 3:**
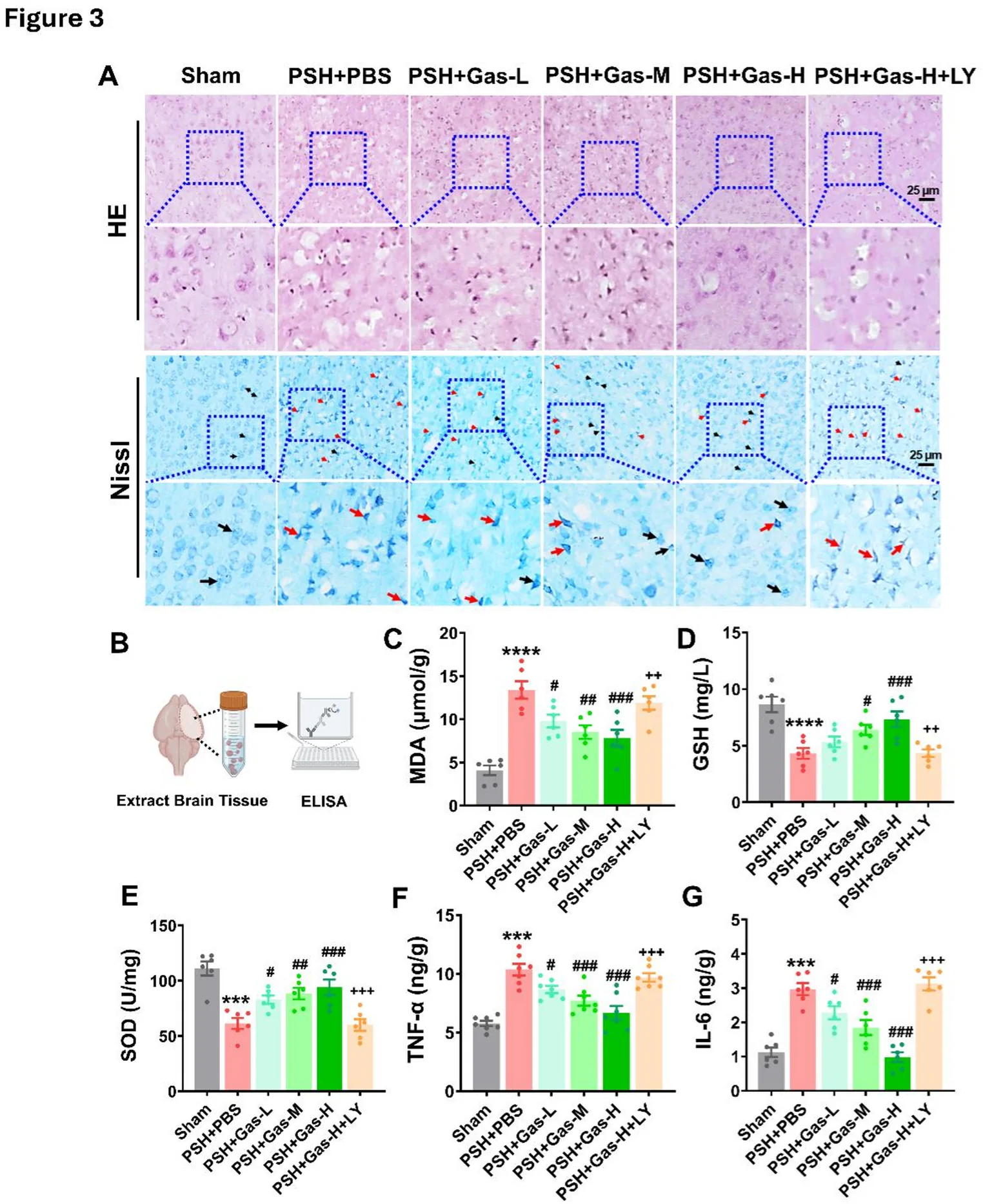
Gastrodin attenuates neuronal pathology and suppresses oxidative stress and neuroinflammation in the ischemic penumbra via the PI3K/Akt pathway. **A** Representative hematoxylin-eosin (HE, upper panel) and Nissl staining (lower panel) of the ischemic penumbra in each group. Black arrows indicate intact neurons with abundant Nissl bodies; red arrows indicate damaged neurons with nuclear pyknosis and Nissl body dissolution. **B** Schematic workflow of brain tissue collection and ELISA detection for oxidative stress and inflammatory markers. **C**-**E** Quantification of oxidative stress indicators in brain tissue homogenates: *N* = 6 rats/group. **C** malondialdehyde (MDA) content, **D** glutathione (GSH) level, and **E** superoxide dismutase (SOD) activity. **F**-**G** Quantification of pro-inflammatory cytokines (**F**) TNF-α and (**G**) IL-6 in brain tissue homogenates. Data are presented as mean ± SEM. ***P < 0.001, **** *P* < 0.0001 vs. Sham; ^#^*P* < 0.05, ^##^*P* < 0.01, ^###^*P* < 0.001 vs. PSH + PBS; ^++^*P* < 0.01, ^+++^*P* < 0.001 vs. PSH + Gas-H group. Statistical comparisons were performed by one-way ANOVA followed by Tukey’s post hoc test

To observe the protective effect of gastrodin on neuronal structure in the ischemic penumbra, we performed HE and Nissl staining on rat brain tissue. As shown in Fig. 3A, the Sham group showed normal neuronal morphology. The PSH model group (PSH + PBS) exhibited typical ischemic changes: tissue edema, disorganized neurons, nuclear pyknosis (HE), and Nissl body dissolution with shrunken somas (Nissl, red arrows). Gastrodin intervention alleviated edema, improved neuronal arrangement, reduced the number of damaged neurons and restored Nissl bodies (black arrows), with the effect more pronounced at higher doses. These protective effects were reversed by LY294002.

Simultaneously, we measured oxidative stress and inflammatory markers in brain tissue homogenates. Compared to the Sham group, the PSH + PBS group showed significantly increased MDA content, decreased GSH and SOD activity (Fig. 3C and E). In addition, the levels of pro-inflammatory cytokines TNF-α and IL-6 were also remarkably upregulated. (Figure 3F and G). Gastrodin treatment dose-dependently reversed these changes, lowering MDA and inflammatory factor levels and restoring GSH and SOD activity (Fig. 3C-G; *P* for trend ≤ 0.0004). The PI3K inhibitor LY294002 abolished these beneficial effects of gastrodin (Fig. 3C-G). These findings indicate that gastrodin suppresses oxidative stress and neuroinflammation via the PI3K/Akt pathway, thereby maintaining the structural integrity of neurons in the ischemic penumbra of PSH rats.

### Gastrodin Enhances the PI3K/Akt Signaling Pathway and Inhibits Downstream Neuronal Apoptosis in PSH Rats

**Fig. 4:**
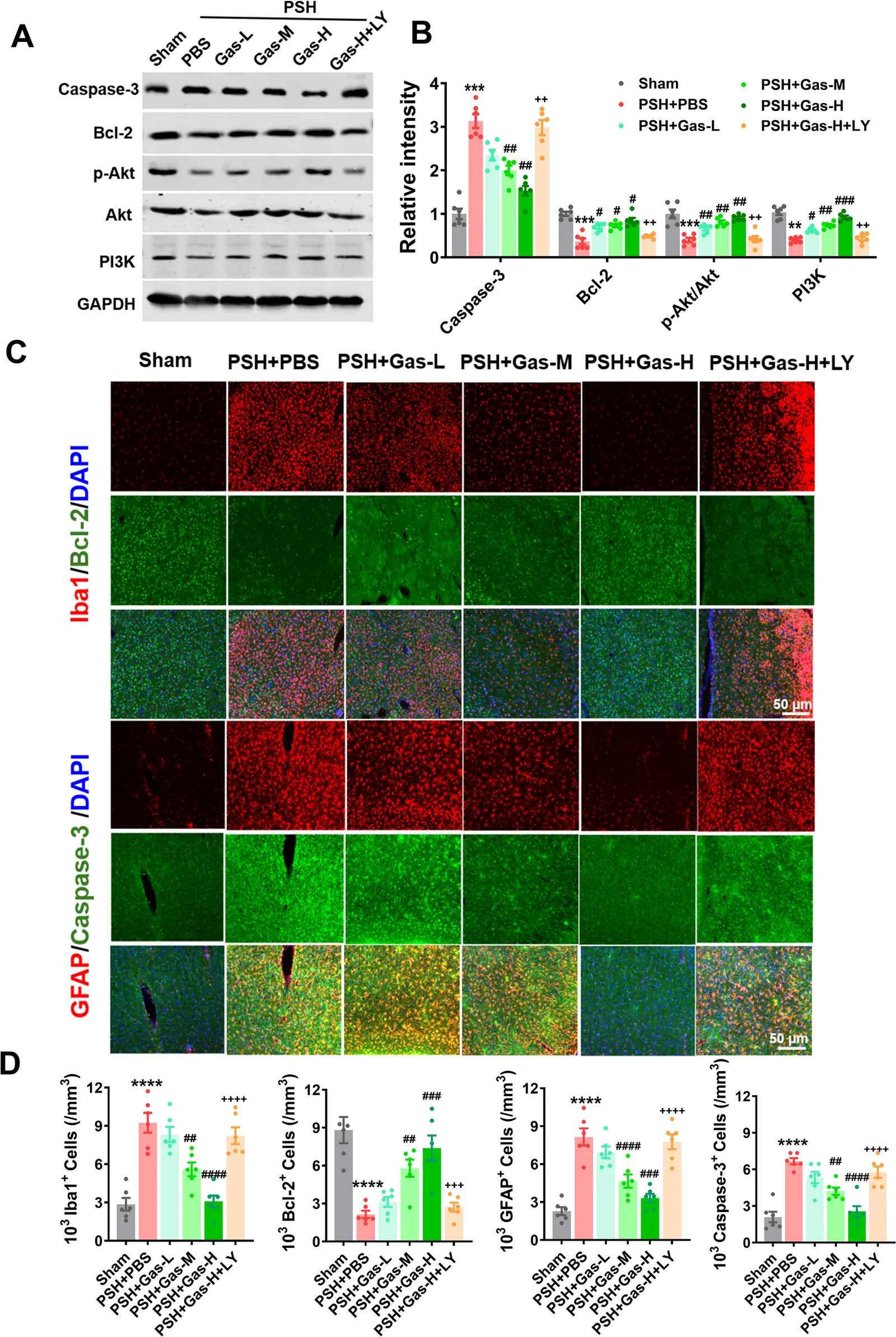
Gastrodin inhibits neuronal apoptosis by enhancing the PI3K/Akt pathway. Representative Western blot bands (left, **A**) and quantitative analysis (right, **B**) of PI3K, p-Akt/Akt ratio, Caspase-3, Bcl-2, and GAPDH in brain tissue from each group. The p-Akt/Akt ratio was calculated to reflect PI3K/Akt pathway activity. *N* = 6 rats/group. **C** Representative immunofluorescence staining for Bcl-2 (green)/Iba1 (red), and Caspase-3 (green)/GFAP (red) in the ischemic penumbra. Nuclei were counterstained with DAPI (blue). **D** Quantitative analysis of the numbers of Bcl-2⁺, Iba1⁺, GFAP⁺, and Caspase-3⁺ cells in the ischemic penumbra. For each animal, five randomly selected fields were averaged to obtain a single value, and data are expressed as cells/mm³. *N* = 6 rats/group. Data are presented as mean ± SEM. **P < 0.01, ***P < 0.001 vs. Sham; ^#^*P* < 0.05, ^##^*P* < 0.01 vs. PSH + PBS group; ^++^*P* < 0.01, ^+++^*P* < 0.001 vs. PSH + Gas-H group. Statistical comparisons were performed by one-way ANOVA followed by Tukey’s post hoc test

To determine whether the PI3K/Akt pathway mediates the neuroprotective and anti-headache effects of gastrodin, we examined the expression of PI3K/Akt pathway and apoptosis-related proteins in brain tissue by Western blot. Compared with the Sham group, the PSH model group showed significantly downregulated PI3K expression and a markedly reduced p-Akt/Akt ratio, indicating ischemia-induced inhibition of PI3K/Akt pathway activity. Concomitantly, the pro-apoptotic protein Caspase-3 was markedly upregulated, whereas the anti-apoptotic protein Bcl-2 was significantly downregulated, indicating enhanced neuronal apoptosis (Fig. 4A and B).

Gastrodin treatment dose-dependently restored PI3K expression and increased the p-Akt/Akt ratio (Fig. 4A and B; *P* for trend < 0.0001), effectively reversing the ischemia-induced inhibition of the PI3K/Akt pathway activity. Concomitantly, gastrodin dose-dependently downregulated Caspase-3 and further upregulated Bcl-2 (Fig. 4A and B; *P* for trend < 0.0001 for both). Immunofluorescence staining further revealed that gastrodin dose-dependently increased the number of Bcl-2⁺ cells and decreased the numbers of Iba1⁺, GFAP⁺ and Caspase-3⁺ cells in the ischemic penumbra (Fig. 4C and D; *P* for trend < 0.0001 for all). All these effects, including the restoration of the p-Akt/Akt ratio, were reversed by the PI3K inhibitor LY294002 (PSH + Gas-H + LY group, Fig. 4A and B), confirming that gastrodin’s actions depend on the PI3K/Akt pathway. Together, these results provide molecular evidence that gastrodin inhibits neuronal apoptosis by restoring the PI3K/Akt pathway, thereby exerting neuroprotective effects in PSH rats.

### Peripheral Blood PI3K/Akt Expression is Downregulated in AIS Patients and Correlates with Post-Stroke Headache Severity

**Fig. 5:**
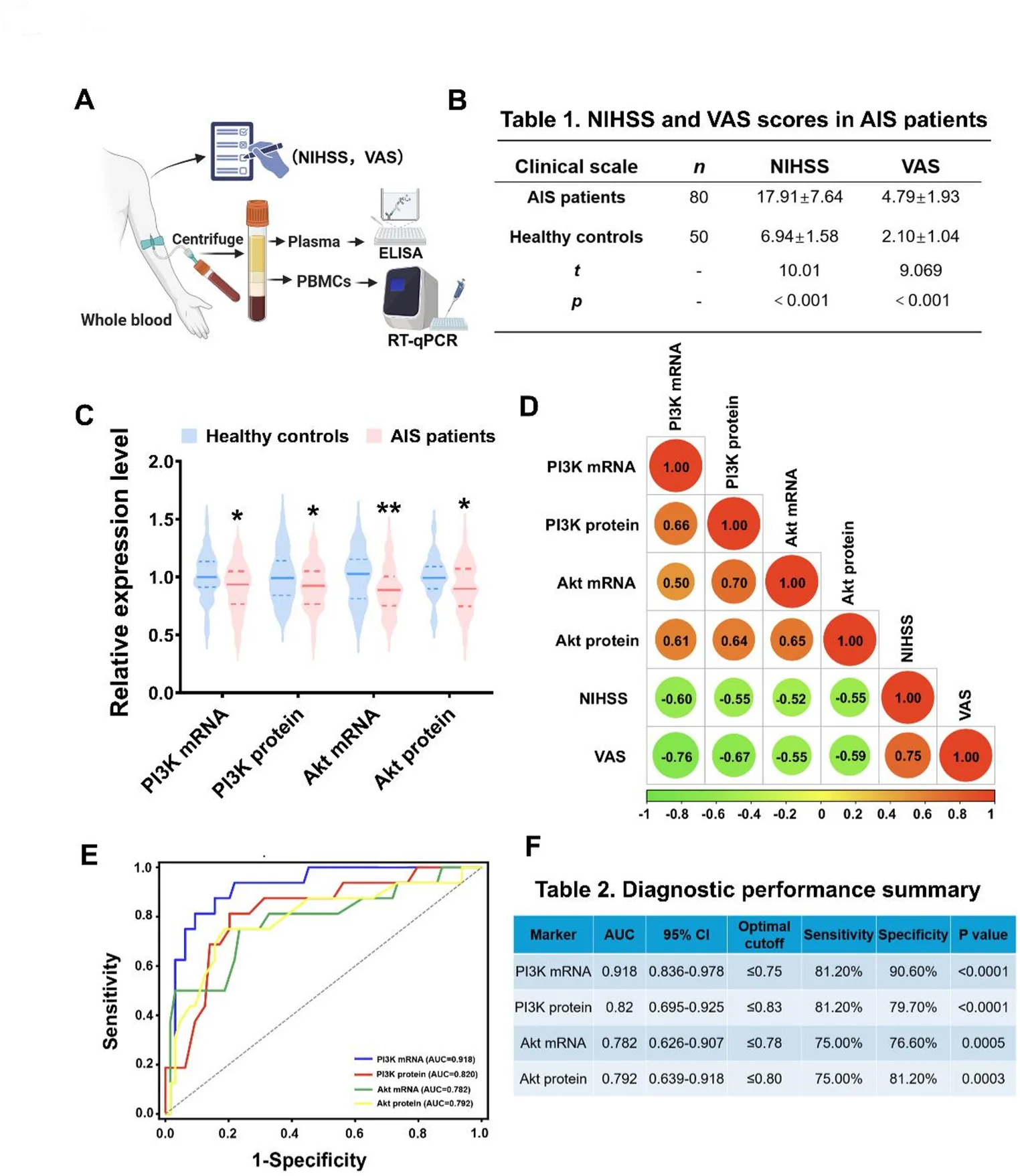
Peripheral blood PI3K/Akt expression is downregulated in AIS patients and inversely correlates with neurological deficit and headache severity. **A** Schematic of the experimental workflow: whole blood was collected, centrifuged to separate plasma and peripheral blood mononuclear cells (PBMCs); plasma was used for ELISA to measure PI3K and Akt protein levels, and PBMCs were used for RT-qPCR to analyze PI3K and Akt mRNA expression. Clinical severity was assessed using the NIHSS and VAS. **B** NIHSS and VAS scores in AIS patients (*n* = 80) and healthy controls (*n* = 50). Data are presented as mean ± SD. NIHSS, National Institutes of Health Stroke Scale; VAS, Visual Analog Scale. **C** Relative expression levels of PI3K and Akt mRNA (PBMCs, RT-qPCR) and protein (plasma, ELISA) in healthy controls (*n* = 50) and AIS patients (*n* = 80). Violin plots show the data distribution; solid lines indicate the median and dashed lines indicate the upper and lower quartiles. *P < 0.05, **P < 0.01 vs. healthy controls (two-tailed Student’s *t*-test). **D** Correlation matrix of PI3K mRNA, PI3K protein, Akt mRNA, Akt protein, NIHSS and VAS. Numbers indicate Pearson correlation coefficients (r) calculated in AIS patients (n = 80). (E-F) ROC curve analysis of peripheral blood PI3K/Akt pathway components for discriminating severe PSH (VAS ≥ 7, *n* = 16) from non-severe PSH (VAS < 7, *n* = 64) in 80 AIS patients. **E** ROC curves for PI3K mRNA, PI3K protein, Akt mRNA, and Akt protein. **F** Summary of AUC, 95% CI, optimal cut-off, sensitivity, specificity, and P value for each marker. Severity stratification was performed post hoc using a clinically established VAS threshold

To validate the clinical relevance of the PI3K/Akt pathway in PSH, this study enrolled 80 AIS patients and 50 healthy controls (Fig. 5A). Patient clinical characteristics are shown in Table 1 (Fig. 5B). Compared to healthy controls, AIS patients showed significantly reduced mRNA and protein expression levels of PI3K and Akt in peripheral blood (Fig. 5C; Table S4, S5; *P* < 0.05, *P* < 0.05, *P* < 0.01, *P* < 0.05), confirming downregulation of this pathway in the acute phase of AIS. Correlation analysis (Fig. 5D) revealed significant positive correlations among PI3K/Akt pathway components (PI3K mRNA vs. PI3K protein: *r* = 0.66; PI3K mRNA vs. Akt mRNA: *r* = 0.50), indicating coordinated expression of pathway components. More importantly, PI3K mRNA, PI3K protein, Akt mRNA, and Akt protein levels were all significantly negatively correlated with NIHSS scores (*r* = −0.60, −0.55, −0.52, −0.55) and with VAS scores (*r* = −0.76, −0.67, −0.55, −0.59). Additionally, NIHSS and VAS scores were strongly positively correlated (*r* = 0.75), indicating a high degree of consistency between neurological deficit severity and post-stroke headache sensitization. In summary, the PI3K/Akt signaling pathway is involved in the pathophysiology of AIS, and its peripheral blood expression level is closely associated with neurological deficits and headache severity, suggesting its potential as a biomarker for assessing AIS severity. To further determine the diagnostic value of the PI3K/Akt pathway, patients were stratified into severe PSH (VAS ≥ 7, *n* = 16) and non-severe PSH (VAS < 7, *n* = 64) subgroups. ROC curve analysis demonstrated that peripheral blood PI3K mRNA discriminated severe from non-severe PSH with an AUC of 0.918 (95% CI 0.836–0.978; sensitivity 81.2%, specificity 90.6% at the optimal cut-off), while PI3K protein, Akt mRNA, and Akt protein yielded AUCs of 0.820, 0.782, and 0.792, respectively (all *P* < 0.001; Fig. 5E and F Table 2). These findings indicate that the peripheral PI3K/Akt pathway, particularly PI3K mRNA, has good-to-excellent diagnostic efficacy for identifying patients with severe PSH.

**Table 1:** NIHSS and VAS scores in AIS patients

Clinical scale	*n*	NIHSS	VAS
AIS patients	80	17.91±7.64	4.79±1.93
Healthy controls	50	6.94±1.58	2.10±1.04
*t*	–	10.01	9.069
*P*	–	< 0.001	< 0.001

NIHSS and VAS scores in AIS patients (n = 80) and healthy controls (n = 50). Data are presented as mean ± SD. NIHSS, National Institutes of Health Stroke Scale; VAS, Visual Analog Scale

**Table 2:** Diagnostic performance summary

Marker	AUC	95% CI	Optimal cutoff	Sensitivity	Specificity	*P* value
PI3K mRNA	0.918	0.836-0.978	≤ 0.75	81.20%	90.60%	< 0.0001
PI3K protein	0.82	0.695-0.925≤0.83	≤ 0.83	81.20%	79.70%	< 0.0001
Akt mRNA	0.782	0.626-0.907	≤ 0.78	75.00%	76.60%	0.0005
Akt protein	0.792	0.626-0.907	≤ 0.80	75.00%	81.20%	0.0003

Summary of AUC, 95% CI, optimal cut-off, sensitivity, specificity, and P value for each marker. Severity stratification was performed post hoc using a clinically established VAS threshold

## Discussion

Acute ischemic stroke remains one of the leading causes of death and long-term disability worldwide [20]. Over the past decade, intravenous thrombolysis combined with mechanical thrombectomy has substantially widened the therapeutic window, yet delayed neuronal death secondary to ischemic injury and post-stroke headache driven by central sensitization remain unresolved clinical problems [42–45]. Agents capable of concurrently modulating multiple injury pathways while crossing the blood-brain barrier are therefore of considerable translational interest.

Existing evidence on gastrodin, the PI3K/Akt pathway, and PSH can be organized into three largely separate lines of investigation, each of which leaves an important question unanswered. First, pharmacological reviews and mechanistic studies have established gastrodin as a multi-target neuroprotectant in ischemic stroke that acts principally through PI3K/Akt activation to limit infarct expansion, oxidative stress, and apoptosis, and, in one recent report, to suppress ferroptosis via the PI3K/AKT/Nrf2 axis [29, 30, 33]. However, these studies uniformly assessed parenchymal injury endpoints (infarct volume, neurological score, cell-death markers) and did not examine any headache- or trigeminal-nociception-related outcome, so whether gastrodin’s neuroprotective action extends to trigeminovascular sensitization was previously untested. Second, the analgesic properties of gastrodin have been characterized almost exclusively in primary headache models: in nitroglycerin-induced migraine, gastrodin suppresses CGRP release and succinate/HIF-1α/TRPM2 signaling in the trigeminal ganglion [31, 32], but these studies were performed in the absence of any ischemic insult and did not implicate PI3K/Akt as the operative mechanism. Third, a role for PI3K/Akt in trigeminal nociception has emerged from vestibular migraine models, in which CXCL10-driven, PI3K-mediated neuroinflammation and TREM2-driven, PI3K/Akt/mTOR-dependent autophagic dysfunction contribute to central sensitization [17, 27], yet these studies were conducted in non-stroke, non-vascular headache paradigms and did not test whether this pathway is pharmacologically targetable by gastrodin or by any clinically relevant agent. Consequently, no prior study had established PI3K/Akt as a mechanistic node common to both ischemic neurodegeneration and trigeminovascular sensitization within a single disease process, tested a pharmacological agent capable of engaging this node to produce concurrent neuroprotective and analgesic benefit, or evaluated whether peripheral PI3K/Akt activity tracks with headache severity in stroke patients. The present study was designed to address these three interconnected gaps by combining a PSH rat model with pharmacological pathway inhibition (LY294002) and a parallel clinical cohort.

Within this framework, upregulation of CGRP and substance P in the trigeminal ganglion [9, 46] and hyperactivation of neurons in the trigeminal nucleus caudalis [47] are recognized as key pathological substrates shared by migraine and post-stroke headache. Our data show that gastrodin’s alleviation of PSH is mechanistically dependent on enhanced PI3K/Akt pathway activity, as reflected by the p-Akt/Akt ratio, and is accompanied by fewer c-Fos-positive neurons in the TNC and lower CGRP and substance P levels in the trigeminal ganglion, effects that were abolished by LY294002, extending this established pathophysiological framework to a PI3K/Akt-defined mechanism in the stroke setting. This mechanism differs from that reported for nitroglycerin-induced migraine, where gastrodin’s antinociceptive action was attributed mainly to peripheral trigeminal ganglion TRPM2/HIF-1α suppression rather than to central PI3K/Akt signaling [31]. Our findings therefore suggest that gastrodin engages distinct, context-dependent mechanisms depending on the underlying headache trigger, and identify restoration of PI3K/Akt pathway activity, rather than peripheral vascular or TRPM2/HIF-1α regulation, as the route by which gastrodin suppresses trigeminovascular sensitization in the stroke setting. This also extends prior work implicating PI3K/Akt in trigeminal central sensitization in non-vascular headache models [17, 27], to a cerebrovascular disease context, and, to our knowledge, provides the first pharmacological demonstration that a PI3K/Akt-activating agent can suppress trigeminal hyperactivation arising from an ischemic rather than a purely nociceptive trigger.

The acute phase of ischemic stroke is also characterized by tightly interlinked neuroinflammation and oxidative stress, in which persistent ischemia triggers a cascade of neuronal necrosis, oxidative stress, neuroinflammation, and apoptosis that culminates in irreversible tissue damage [48–50], and PI3K/Akt signaling has been identified as a central hub regulating cell survival and tissue-microenvironment homeostasis in this setting [51, 52]. With respect to neuroprotection, gastrodin dose-dependently reduced infarct volume, improved neurological scores, and restored cerebral blood flow, while lowering MDA and pro-inflammatory cytokines, restoring SOD/GSH, and shifting the Bcl-2/Caspase-3 balance. These findings are broadly consistent with previous reports describing gastrodin as an antioxidant, anti-inflammatory, and anti-apoptotic agent in ischemic stroke [29, 30], and align with the wider literature establishing PI3K/Akt as a convergent survival hub for natural-compound neuroprotectants, including quercetin, B. glomerulata extract, and Gastrodia elata extract itself, each of which limits apoptosis and/or oxidative injury through PI3K/Akt-dependent mechanisms [18, 19, 21, 24, 26]. Our study extends this literature in two respects. First, whereas Wang et al. emphasized ferroptosis suppression via the PI3K/AKT/Nrf2 axis as the dominant protective mechanism of Gastrodia elata extract [33], our data implicate a parallel route centered on suppression of microglial activation (Iba1), astrocytic apoptosis (GFAP/Caspase-3), and the classical Bcl-2/Caspase-3 apoptotic axis, indicating that PI3K/Akt-dependent neuroprotection by Gastrodia elata-derived compounds may operate through more than one cell-death pathway depending on dose, formulation, and injury context. Second, and more importantly, none of these prior reports evaluated a pain or trigeminal phenotype alongside parenchymal injury. By assessing both outcomes within the same MCAO-PSH model and reversing both with the same PI3K inhibitor, our data provide direct evidence that PI3K/Akt functions as a shared molecular hub linking ischemic parenchymal injury to trigeminovascular sensitization, rather than as two independently regulated processes that happen to respond to the same drug. Gastrodin showed a dose-dependent effect across all the endpoints examined (linear trend *P* ≤ 0.0004; Table S3). For the periorbital mechanical withdrawal threshold, the response tended to plateau between 100 and 200 mg/kg (significant non-linear component, *P* = 0.0002), suggesting that the maximal effect may be approached at the higher doses. Because only three doses (50, 100 and 200 mg/kg) were tested and none above 200 mg/kg was included, 200 mg/kg should be regarded as the most effective dose tested rather than the optimum, and the optimal effective dose range could not be defined. Non-monotonic responses to gastrodin have been reported in other settings [32, 53], and biphasic dose-responses have been described for herbal compounds [54]; a finer and wider dose gradient is therefore warranted in future studies.

From a translational standpoint, peripheral blood PI3K and Akt levels were significantly lower in AIS patients than in healthy controls and were inversely correlated with both neurological deficit (NIHSS) and headache severity (VAS), with PI3K mRNA showing the best discriminative performance for severe PSH (AUC = 0.918). Peripheral blood biomarkers have previously been explored for other post-stroke complications, such as blood-brain-barrier disruption and cognitive impairment, but, to our knowledge, no prior study has linked peripheral PI3K/Akt pathway activity to post-stroke headache specifically. This finding therefore provides the first clinical evidence connecting the PI3K/Akt axis identified in our preclinical model to a headache-relevant phenotype in AIS patients, and nominates peripheral PI3K/Akt, particularly PI3K mRNA, as a candidate biomarker for identifying patients at risk of severe PSH. Nonetheless, given the post hoc severity stratification, the relatively small severe-PSH subgroup (*n* = 16), and the single-center design, these findings should be regarded as hypothesis-generating pending external validation in larger, prospective, multicenter cohorts.

This study has several limitations. First, we mainly used a pharmacological inhibitor (LY294002) to verify pathway dependence; a LY294002-only control group was not included, so the possibility of independent effects of LY294002 on PSH and ischemic brain injury cannot be fully excluded. Future studies incorporating a LY294002-only control and/or cell-specific PI3K or Akt knockout mice are required for rigorous genetic confirmation. Moreover, LY294002 is a reversible, non-selective PI3K inhibitor that only partially suppresses Akt phosphorylation [55–57]; as it was co-administered only with 200 mg/kg gastrodin, whether lower doses are equally PI3K/Akt-dependent, or whether incomplete inhibition yields partial effects, remains unknown. Second, we only explored the apoptotic mechanism downstream of PI3K/Akt; whether this pathway also participates in autophagy or synaptic plasticity remains to be investigated. Third, while we have demonstrated the therapeutic efficacy of gastrodin in animal models, the optimal administration route, treatment timing, and long-term efficacy of gastrodin for AIS and PSH still require validation through large-scale clinical trials. Fourth, detailed clinical information, including headache duration, migraine history, thrombolytic/thrombectomy history, and concomitant medications, was not recorded, and confounding factors were not matched; these omissions may introduce residual confounding, and the clinical correlation findings should therefore be regarded as exploratory. Future validation in larger, multicenter prospective cohorts with systematic collection of these variables and appropriate adjustment for confounders is warranted.

In conclusion, by directly comparing our findings against three previously separate literatures on gastrodin, PI3K/Akt signaling, and PSH, we show that gastrodin ameliorates both post-stroke headache and persistent ischemia-induced brain injury by enhancing the PI3K/Akt signaling pathway, thereby identifying PI3K/Akt as a shared mechanistic node linking ischemic neurodegeneration and trigeminovascular sensitization. This closes a previously unaddressed gap between the stroke-neuroprotection and headache-mechanism literatures, and provides a new theoretical basis and candidate therapeutic target and biomarker for the precise intervention of AIS and post-stroke headache.

## Supplementary Information

Below is the link to the electronic supplementary material.

Supplementary Material 1

Supplementary Material 2

Supplementary Material 3

## Data Availability

Data supporting the findings of this study are available from the corresponding author upon reasonable request.

## Abbreviations

PSH: Post-stroke headache
AIS: Acute ischemic stroke
MCAO: Middle cerebral artery occlusion
TVS: Trigeminal vascular system
CGRP: Calcitonin gene-related peptide
PI3K/Akt: Phosphoinositide 3-kinase/Protein Kinase B
TNC: Trigeminal nucleus caudalis
Gas: Gastrodin
TTC: 2,3,5-triphenyltetrazolium chloride
CBF: Cerebral blood flow
HE: Hematoxylin and Eosin staining
MDA: Malondialdehyde
GSH: Glutathione
SOD: Superoxide dismutase
TNF-α: Tumor necrosis factor-alpha
IL-6: Interleukin-6
ELISA: Enzyme-linked immunosorbent assay
NIHSS: National institutes of health stroke scale
VAS: Visual analog scale
