# Supplementary material for: Gastrodin Alleviates Post-Stroke Headache and Neuronal Injury via Activating the PI3K/Akt Pathway
Source: Neurochem Res. 2026 Nov 12;51(6):329. doi: 10.1007/s11064-026-04932-2 (PMC13759594; doi:10.1007/s11064-026-04932-2)
Supplement: Supplementary file 1 [file 11064_2026_4932_MOESM1_ESM.docx]

**Supplementary material**

**Table S1 24-h model success rate**

| Animal Number | MCAO | | | | | |
| --- | --- | --- | --- | --- | --- | --- |
|  | non-PSH | PSH+PBS | PSH+Gas-L | PSH+Gas-M | PSH+Gas-H | PSH+Gas-H+LY |
| Total rats | 36 | 24 | 24 | 24 | 22 | 25 |
| Deaths within 24 h | 4 | 3 | 2 | 3 | 2 | 2 |
| 24 h survivors | 32 | 21 | 22 | 21 | 20 | 23 |
| Animals with inadequate CBF | 2 | 0 | 1 | 1 | 0 | 1 |
| Zea-Longa score = 0 | 1 | 0 | 0 | 0 | 0 | 0 |
| Zea-Longa score = 4 | 0 | 0 | 0 | 0 | 0 | 0 |
| Animals enrolled on day 1 | 29 | 21 | 21 | 20 | 20 | 22 |
| Modeling success rate | 29/36 (80.56%) | 21/24 (87.50%) | 21/24 (87.50%) | 20/24 (83.33%) | 20/22 (90.91%) | 22/25  (88.00%) |
| Total Modeling success rate | 133/155 (85.81%) | | | | | |

**Table S2 Animal mortality**

| time-point | Dead rats / Total rats | | | | | | |
| --- | --- | --- | --- | --- | --- | --- | --- |
|  | Sham | MCAO | | | | | |
|  |  | non-PSH | PSH+PBS | PSH+Gas-L | PSH+Gas-M | PSH+Gas-H | PSH+Gas-H+LY |
| 0~3d | 0/18 | 5/36 | 3/24 | 3/24 | 3/24 | 2/22 | 2/25 |
| 3~7d | 0/18 | 2/31 | 2/21 | 2/21 | 1/21 | 1/20 | 3/23 |
| 7~14d | 0/18 | 2/29 | 1/19 | 0/19 | 1/20 | 1/19 | 1/20 |
| 14~21d | 0/18 | 0/27 | 0/18 | 0/19 | 0/19 | 0/18 | 0/19 |
| Total | 0/18 (0%) | 9/36 (25%) | 6/24 (25.0%) | 5/24 (20.83%) | 5/24 (20.83%) | 4/22 (18.18%) | 6/25  (24.0%) |
| Total | 35/173 (20.23%) | | | | | | |

**Table S3 Dose-response trend analysis for all examined endpoints**

| Figure | Endpoint | n/group | Slope (95% CI) | Linear trend P | Non-linear P |
| --- | --- | --- | --- | --- | --- |
| 1E | c-Fos⁺ cells | 5 | -19.84  (-23.41, -16.27) | <0.0001 | 0.1446 |
| 1G | CGRP | 6 | -0.2527  (-0.3583, -0.1470) | <0.0001 | 0.9197 |
| 1G | Substance P | 6 | -0.3332  (-0.4416, -0.2247) | <0.0001 | 0.9926 |
| 1H | Withdrawal threshold | 6 | 1.740  (1.515, 1.965) | <0.0001 | 0.0002 |
| 2A | Zea-Longa score | 12 | -0.4833  (-0.6474, -0.3192) | <0.0001 | 0.5492 |
| 2B | Rotarod latency | 12 | 25.33  (19.48, 31.17) | <0.0001 | 0.7605 |
| 2D | CBF | 7 | 10.93  (8.976, 12.88) | <0.0001 | 0.7027 |
| 2F | Infarct volume | 6 | -6.566  (-7.369, -5.762) | <0.0001 | 0.1739 |
| 3C | MDA | 6 | -1.803  (-2.618, -0.9882) | 0.0002 | 0.2572 |
| 3D | GSH | 6 | 1.010  (0.5163, 1.504) | 0.0004 | 0.9962 |
| 3E | SOD | 6 | 10.36  (5.331, 15.38) | 0.0003 | 0.2924 |
| 3F | TNF-α | 7 | -1.200  (-1.625, -0.7743) | <0.0001 | 0.7226 |
| 3G | IL-6 | 6 | -0.6398  (-0.8122, -0.4675) | <0.0001 | 0.6409 |
| 4B | Caspase-3 | 6 | -0.5160  (-0.6341, -0.3979) | <0.0001 | 0.3099 |
| 4B | Bcl-2 | 6 | 0.1470  (0.09801, 0.1960) | <0.0001 | 0.0578 |
| 4B | p-Akt/Akt | 6 | 0.1680  (0.1301, 0.2059) | <0.0001 | 0.1624 |
| 4B | PI3K | 6 | 0.1697  (0.1427, 0.1968) | <0.0001 | 0.3268 |
| 4D | Iba1⁺ cells | 6 | -2.122  (-2.688, -1.556) | <0.0001 | 0.3384 |
| 4D | Bcl-2⁺ cells | 6 | 1.839  (1.233, 2.445) | <0.0001 | 0.5784 |
| 4D | GFAP⁺ cells | 6 | -1.678  (-2.158, -1.199) | <0.0001 | 0.6853 |
| 4D | Caspase-3⁺ cells | 6 | -1.350  (-1.697, -1.003) | <0.0001 | 0.8284 |

**Table S4 Record of Molecular Markers and Functional Scores in AIS patients**

| Patient | PI3K mRNA | PI3K protein | Akt mRNA | Akt protein | NIHSS | VAS |
| --- | --- | --- | --- | --- | --- | --- |
| 1 | 0.81 | 0.92 | 0.90 | 0.83 | 18 | 6 |
| 2 | 1.06 | 1.03 | 1.10 | 1.06 | 10 | 3 |
| 3 | 0.94 | 1.08 | 1.06 | 0.90 | 14 | 5 |
| 4 | 0.85 | 0.90 | 0.89 | 0.87 | 16 | 5 |
| 5 | 0.94 | 0.97 | 0.97 | 0.68 | 17 | 5 |
| 6 | 1.16 | 1.27 | 1.24 | 1.18 | 8 | 2 |
| 7 | 1.26 | 1.31 | 1.27 | 1.23 | 7 | 2 |
| 8 | 1.28 | 1.27 | 1.25 | 1.17 | 10 | 2 |
| 9 | 0.99 | 0.75 | 0.64 | 0.91 | 18 | 6 |
| 10 | 0.89 | 0.93 | 0.97 | 1.07 | 15 | 5 |
| 11 | 0.92 | 1.14 | 0.92 | 1.15 | 23 | 6 |
| 12 | 0.91 | 1.10 | 0.71 | 0.83 | 11 | 3 |
| 13 | 0.98 | 1.05 | 1.13 | 1.09 | 18 | 4 |
| 14 | 0.87 | 1.05 | 1.08 | 1.04 | 15 | 5 |
| 15 | 1.04 | 1.25 | 0.92 | 0.93 | 24 | 3 |
| 16 | 0.50 | 0.85 | 1.03 | 0.69 | 11 | 5 |
| 17 | 0.75 | 0.71 | 1.13 | 1.21 | 13 | 8 |
| 18 | 1.08 | 1.05 | 1.17 | 1.13 | 10 | 3 |
| 19 | 1.08 | 1.03 | 1.10 | 1.17 | 10 | 3 |
| 20 | 1.04 | 1.14 | 1.08 | 1.07 | 11 | 3 |
| 21 | 0.65 | 0.76 | 0.77 | 0.90 | 21 | 7 |
| 22 | 0.98 | 0.97 | 0.90 | 0.90 | 13 | 4 |
| 23 | 1.02 | 0.90 | 0.85 | 1.07 | 16 | 5 |
| 24 | 0.45 | 0.49 | 0.54 | 0.61 | 25 | 8 |
| 25 | 0.94 | 0.98 | 0.96 | 1.09 | 16 | 5 |
| 26 | 0.67 | 0.76 | 0.78 | 0.74 | 23 | 7 |
| 27 | 1.18 | 1.25 | 1.10 | 1.09 | 7 | 2 |
| 28 | 0.77 | 1.05 | 0.92 | 1.01 | 12 | 4 |
| 29 | 1.06 | 1.15 | 0.89 | 0.90 | 10 | 3 |
| 30 | 0.78 | 0.81 | 0.77 | 0.71 | 20 | 6 |
| 31 | 1.13 | 1.12 | 1.08 | 1.04 | 8 | 2 |
| 32 | 1.11 | 1.17 | 1.01 | 1.07 | 9 | 3 |
| 33 | 0.82 | 0.93 | 0.89 | 0.90 | 20 | 6 |
| 34 | 1.12 | 1.03 | 0.92 | 1.02 | 12 | 3 |
| 35 | 1.12 | 1.12 | 1.24 | 1.31 | 10 | 2 |
| 36 | 0.60 | 0.58 | 0.59 | 0.46 | 26 | 8 |
| 37 | 1.05 | 0.90 | 0.89 | 0.91 | 16 | 6 |
| 38 | 0.98 | 1.00 | 0.92 | 1.01 | 13 | 4 |
| 39 | 1.23 | 1.08 | 1.25 | 1.20 | 7 | 2 |
| 40 | 0.78 | 0.98 | 0.89 | 0.90 | 22 | 6 |
| 41 | 0.60 | 0.81 | 0.77 | 0.71 | 24 | 7 |
| 42 | 1.05 | 1.10 | 0.99 | 1.13 | 16 | 3 |
| 43 | 0.47 | 0.44 | 0.57 | 0.77 | 36 | 9 |
| 44 | 0.61 | 0.76 | 1.01 | 0.61 | 28 | 7 |
| 45 | 0.71 | 0.75 | 0.63 | 0.72 | 29 | 7 |
| 46 | 1.12 | 1.15 | 1.29 | 1.18 | 10 | 2 |
| 47 | 1.01 | 0.80 | 0.97 | 1.07 | 12 | 4 |
| 48 | 0.81 | 0.88 | 0.90 | 0.98 | 29 | 6 |
| 49 | 0.99 | 1.08 | 0.97 | 0.99 | 16 | 4 |
| 50 | 0.82 | 0.71 | 0.78 | 0.61 | 28 | 7 |
| 51 | 1.22 | 1.32 | 0.90 | 1.23 | 9 | 2 |
| 52 | 0.74 | 0.92 | 0.85 | 0.90 | 24 | 6 |
| 53 | 1.09 | 0.93 | 0.73 | 0.82 | 21 | 6 |
| 54 | 0.58 | 0.76 | 0.63 | 0.55 | 31 | 7 |
| 55 | 0.87 | 0.95 | 0.89 | 0.68 | 25 | 6 |
| 56 | 0.95 | 1.03 | 0.71 | 0.98 | 26 | 4 |
| 57 | 0.89 | 0.88 | 0.85 | 0.90 | 25 | 5 |
| 58 | 0.98 | 0.93 | 0.66 | 0.90 | 15 | 2 |
| 59 | 1.26 | 0.95 | 0.85 | 1.39 | 13 | 2 |
| 60 | 0.81 | 0.88 | 0.84 | 0.88 | 17 | 6 |
| 61 | 0.61 | 0.71 | 0.61 | 0.68 | 34 | 8 |
| 62 | 0.72 | 1.03 | 1.37 | 1.13 | 22 | 3 |
| 63 | 0.58 | 0.98 | 0.84 | 0.66 | 20 | 8 |
| 64 | 1.25 | 0.75 | 0.75 | 0.57 | 16 | 5 |
| 65 | 0.94 | 0.63 | 0.84 | 1.02 | 21 | 4 |
| 66 | 0.79 | 0.88 | 0.59 | 0.90 | 9 | 7 |
| 67 | 0.88 | 0.69 | 0.66 | 0.66 | 36 | 6 |
| 68 | 0.92 | 0.86 | 0.75 | 1.12 | 15 | 5 |
| 69 | 0.77 | 0.61 | 0.78 | 0.72 | 22 | 6 |
| 70 | 0.44 | 0.69 | 0.66 | 0.82 | 20 | 5 |
| 71 | 0.95 | 0.83 | 0.61 | 1.07 | 35 | 8 |
| 72 | 0.68 | 0.71 | 0.63 | 0.74 | 24 | 6 |
| 73 | 0.71 | 1.10 | 0.92 | 0.80 | 38 | 8 |
| 74 | 0.69 | 0.71 | 0.50 | 0.80 | 10 | 3 |
| 75 | 1.01 | 0.76 | 0.80 | 0.44 | 21 | 4 |
| 76 | 1.04 | 0.86 | 0.80 | 0.80 | 13 | 2 |
| 77 | 0.96 | 0.78 | 0.77 | 0.83 | 16 | 4 |
| 78 | 0.98 | 0.81 | 0.82 | 0.87 | 12 | 4 |
| 79 | 0.91 | 0.86 | 0.64 | 0.74 | 15 | 5 |
| 80 | 1.11 | 0.92 | 0.82 | 0.88 | 15 | 3 |

**Table S5 Record of Molecular Markers and Functional Scores in healthy controls**

| ID | PI3K mRNA | PI3K protein | Akt mRNA | Akt protein | NIHSS | VAS |
| --- | --- | --- | --- | --- | --- | --- |
| 1 | 1.19 | 1.25 | 1.04 | 0.98 | 6 | 1 |
| 2 | 0.88 | 0.86 | 1.01 | 1.23 | 7 | 2 |
| 3 | 0.94 | 1.32 | 0.61 | 1.07 | 6 | 2 |
| 4 | 1.09 | 0.97 | 0.78 | 0.96 | 6 | 2 |
| 5 | 0.94 | 1.08 | 0.92 | 1.45 | 6 | 2 |
| 6 | 0.92 | 1.14 | 1.11 | 1.20 | 7 | 1 |
| 7 | 0.64 | 0.78 | 0.85 | 1.01 | 12 | 3 |
| 8 | 1.02 | 1.32 | 0.96 | 0.85 | 6 | 2 |
| 9 | 0.57 | 0.78 | 1.46 | 0.93 | 7 | 0 |
| 10 | 1.01 | 0.68 | 0.97 | 0.85 | 7 | 2 |
| 11 | 1.08 | 1.24 | 1.08 | 0.98 | 5 | 2 |
| 12 | 0.78 | 0.95 | 1.03 | 0.99 | 7 | 5 |
| 13 | 0.78 | 0.81 | 1.29 | 0.87 | 8 | 2 |
| 14 | 1.01 | 0.71 | 1.15 | 0.90 | 9 | 2 |
| 15 | 1.04 | 1.02 | 0.54 | 1.02 | 8 | 3 |
| 16 | 1.09 | 1.22 | 1.24 | 1.09 | 8 | 3 |
| 17 | 0.81 | 1.10 | 0.82 | 0.60 | 5 | 1 |
| 18 | 1.22 | 0.69 | 0.70 | 1.23 | 7 | 3 |
| 19 | 1.13 | 1.12 | 0.92 | 1.02 | 9 | 2 |
| 20 | 1.05 | 0.85 | 1.34 | 1.20 | 7 | 2 |
| 21 | 1.01 | 0.86 | 1.32 | 1.09 | 6 | 4 |
| 22 | 0.96 | 0.85 | 1.13 | 0.99 | 4 | 3 |
| 23 | 1.21 | 1.37 | 0.92 | 0.82 | 7 | 3 |
| 24 | 1.18 | 0.90 | 0.78 | 0.69 | 7 | 1 |
| 25 | 1.47 | 0.92 | 1.03 | 0.99 | 7 | 2 |
| 26 | 1.29 | 0.63 | 1.24 | 1.04 | 6 | 3 |
| 27 | 1.30 | 1.42 | 1.03 | 0.91 | 6 | 3 |
| 28 | 0.77 | 0.83 | 0.96 | 1.02 | 5 | 1 |
| 29 | 1.12 | 0.92 | 0.82 | 1.15 | 6 | 3 |
| 30 | 0.98 | 1.34 | 1.36 | 0.96 | 8 | 1 |
| 31 | 1.09 | 0.76 | 1.25 | 0.90 | 6 | 4 |
| 32 | 0.94 | 0.97 | 0.77 | 1.09 | 6 | 0 |
| 33 | 0.79 | 1.00 | 1.11 | 0.85 | 5 | 2 |
| 34 | 0.85 | 1.27 | 0.61 | 0.98 | 6 | 3 |
| 35 | 1.16 | 1.08 | 1.17 | 1.18 | 13 | 1 |
| 36 | 1.22 | 1.08 | 1.13 | 1.06 | 7 | 2 |
| 37 | 0.99 | 1.02 | 0.71 | 0.90 | 7 | 2 |
| 38 | 0.98 | 1.22 | 1.17 | 1.06 | 7 | 1 |
| 39 | 0.82 | 0.68 | 1.27 | 0.60 | 7 | 2 |
| 40 | 0.92 | 1.00 | 1.08 | 1.40 | 6 | 0 |
| 41 | 0.79 | 1.53 | 0.77 | 0.98 | 9 | 3 |
| 42 | 1.13 | 0.86 | 1.32 | 1.07 | 7 | 3 |
| 43 | 0.92 | 1.00 | 1.04 | 0.65 | 8 | 1 |
| 44 | 1.13 | 1.00 | 0.99 | 0.99 | 7 | 3 |
| 45 | 0.94 | 1.08 | 0.68 | 1.10 | 9 | 3 |
| 46 | 0.95 | 0.68 | 0.80 | 1.12 | 6 | 1 |
| 47 | 1.11 | 0.78 | 0.85 | 1.10 | 7 | 2 |
| 48 | 0.94 | 1.15 | 1.06 | 0.82 | 6 | 2 |
| 49 | 0.52 | 0.92 | 1.11 | 1.15 | 6 | 2 |
| 50 | 1.33 | 0.98 | 0.71 | 0.93 | 7 | 2 |
